# Croupal Diphtheria

**Published:** 1859-08

**Authors:** F. H. Evans

**Affiliations:** Aberdeen, Mississippi


### ARTICLE VIII.

*Croupal Diphtheria.* By F. H. Evans, M. D., of Aberdeen, Mississippi.

Diphtheritic Angina has been abundantly discussed for the past nine months in Europe, particularly in London.

That the affection is local, is a fact placed beyond the shadow of a doubt; tnat all constitutional or functional disturbances in attendance, are either symptomatic of, or the consequences of the local disorder, is a fact fixed beyond question.

Then the proposition to abort the disease, by local treatment, is certainly rational. The difficulties are the sudden debut of the disease, and its rapid development, usually descending below the pharynx before medical assistance is obtained. Local applications of the perchloride of iron, the nitrate of silver, and the strong acids; the internal administration of the perchloride and sesquichlorides of iron, the chlorate of potassa and emetics of the tartrate of antimony and potassa, of ipecacuanha, and of sulphate of copper have all been recommended and adopted.

I propose nothing new in this article, but as I have seen no full exposition of the manner of application of the above remedies, I propose my plan which has brought to a successful termination 34 of 35 cases which have come under my care during the past six weeks.

1st. *Cauterization.*—When the disease is confined to the pharynx I employ the nitrate of silver in pencil. When it has progressed below the pharynx I employ the sponge probang, imbibed in a solution of the nitrate of silver after the following formula: Argenti nitras, ʒj; aqua, f℥j. I make these applications at least once every 12 hours, until the disease begins to lose its force. As the disease subsides I protract the period of cauterization, but never discontinue them entirely until the fever subsides.

2d. *Emetics.*—I effect emesis by ipecacuanha 3 times a day; if it debilitates the patient much, or produces catharsis, (which is not often the case,) I employ in its stead the sulphate of copper. I continue the emetics until the fever vanishes.

3d. *Internal or Blood Agencies.*—I administer the chlorate of potassa in doses of from grs. x. to ℈j., 3 times a day. I administer the sesquichloride of iron in doses of from 5 to 30 drops every 6 hours.

4th. I order a tepid bath for half hour every day, and a hot foot bath containing mustard or capsicum every night at bed-time.

REMARKS.—Each of the 35 cases mentioned, was well developed; the tonsils were hypertrophied; the pseudo-membranous deposition were abundant; the fever was intense, and the cough characteristtc and frequent. The fatal case was pronounced convalescent, and discharged at one time. The pharynx presented a normal appearance; the fever was rapidly declining, and the cough was almost silent. I was called again to see the case after a lapse of 5 or 6 days. I could not consider it a relapse, for the larynx was still healthy. I am persuaded, that pseudo-membranous formation was continually going on in the respiratory organs, which was moderated so much by the treatment as to delude me, returning with renewed force under the suspension of the remedies.

The duration of the disease was in these cases from 7 to 11 days. The disease was confined to neither sex nor age.

